# Synergistic Effects of Auranofin and Lysozyme Against *Staphylococcus Aureus*

**DOI:** 10.1007/s12602-026-10983-2

**Published:** 2026-03-17

**Authors:** Qi Zhang, Yang Yang, Shuqi Li, Jiachi Chiou, Qian Zhao

**Affiliations:** 1 Department of Applied Biology and Chemical Technology, The Hong Kong Polytechnic University, Hong Kong, China; 2 Centre for Eye and Vision Research (CEVR), 17W Hong Kong Science Park, Hong Kong, China; 3 Department of Food Science and Nutrition, Research Institute for Future Food, The Hong Kong Polytechnic University, Hong Kong, China

**Keywords:** Antimicrobial resistance, Auranofin, Lysozyme, Combination therapy, WalR

## Abstract

Antimicrobial-resistant bacteria, especially methicillin-resistant *Staphylococcus aureus* (MRSA) are posing a significant public health challenge. Traditional antibacterial treatments rely on external antimicrobials or their combinations to kill pathogens, yet the potential of enhancing the body’s own innate immune mechanisms as an alternative strategy has remained underexplored. As one of the most representative immune molecules, lysozyme, a widely present self-secreted antimicrobial enzyme, serves as a typical daily safeguard against infections in tissues, especially in the eyes, oral cavity and skin. Nonetheless, this defense is showing a declining trend in efficacy against MRSA. Meanwhile, the anti-rheumatic drug auranofin is being repurposed for its antibacterial effects, notably against Gram-positive bacteria. This study demonstrated that a synergistic combination of auranofin and lysozyme at physiological concentration could inhibit diverse *Staphylococcus aureus* growth and mitigate the risk of inducing drug-resistant mutation during continuous passages. Furthermore, this combination significantly reduced biofilm biomass. Morphological and proteomic analyses revealed that auranofin synergized with lysozyme by promoting cell wall autolysis through dysregulation of the WalRK regulatory system. This was accompanied by elevated reactive oxygen species (ROS) and depleted adenosine triphosphate (ATP). The therapeutic relevance of this synergy was confirmed in a cell-based infection model. Overall, our findings highlight the potential of auranofin as a novel adjuvant to potentiate the innate immune function of lysozyme, offering a promising approach to combat drug-resistant bacterial infections by reinvigorating the host’s inherent defense mechanisms.

**Supplementary Information:**

The online version contains supplementary material available at 10.1007/s12602-026-10983-2.

## Introduction

Antimicrobial is regarded as one of the most successful drugs developed in human history because it has saved countless lives [1]. Currently, bacterial infections are still primarily treated with antimicrobial therapy, where drug selection depends on multiple factors including the pathogen type, infection characteristics, patient health status, and regional antimicrobial resistance patterns [2, 3]. With the overuse and misuse of antimicrobial, there are increasing reports of antimicrobial-resistant (AMR) superbugs [4], such as MRSA which are highly prevalent in hospital-associated bacterial infections [5, 6]. Besides, the frequent use of medical assistive devices such as lenses promotes bacterial biofilm formation, making it even more challenging to eradicate bacteria on these non-living surfaces [7, 8]. Biofilms consist of bacteria and a secreted matrix of extracellular polymeric substances (EPSs) on living or non-living surfaces [7]. Once formed, bacteria within biofilms can share nutrients and antimicrobial resistance genes, and desensitize many antimicrobials [3, 7, 9]. These challenges underscore the urgency of finding more effective strategies to address these infections.

In contrast to conventional strategies that rely solely on external antimicrobials, enhancing the body’s own innate immune mechanisms represents a promising yet underexplored alternative [10]. This approach aims to reinvigorate endogenous host defenses, offering a potential means to combat resistant infections while reducing selective pressure that drives resistance [1, 11]. Lysozyme, an abundant secretion from glands (≥ 1 mg/ml in adult tears), is recognized as a typical antimicrobial defense that protects skins, orals and eyes from bacterial infections [11]. It can kill bacteria by hydrolyzing 1,4-β-linkages between N-acetyl-muramic acid and N-acetyl-D-glucosamine residues in the peptidoglycan of bacterial cell walls [11]. As a key component of the innate immune system, lysozyme serves as a first-line defense against bacterial invasion at mucosal surfaces [11]. Despite the presence of lysozyme, both tear and saliva are often ineffective in treating certain bacterial infections, especially those caused by MRSA [11], suggesting that its standalone activity is often insufficient to eradicate these resistant pathogens and highlighting the necessity to potentiate its antibacterial efficacy within innate immune system.

**Fig. 1:**
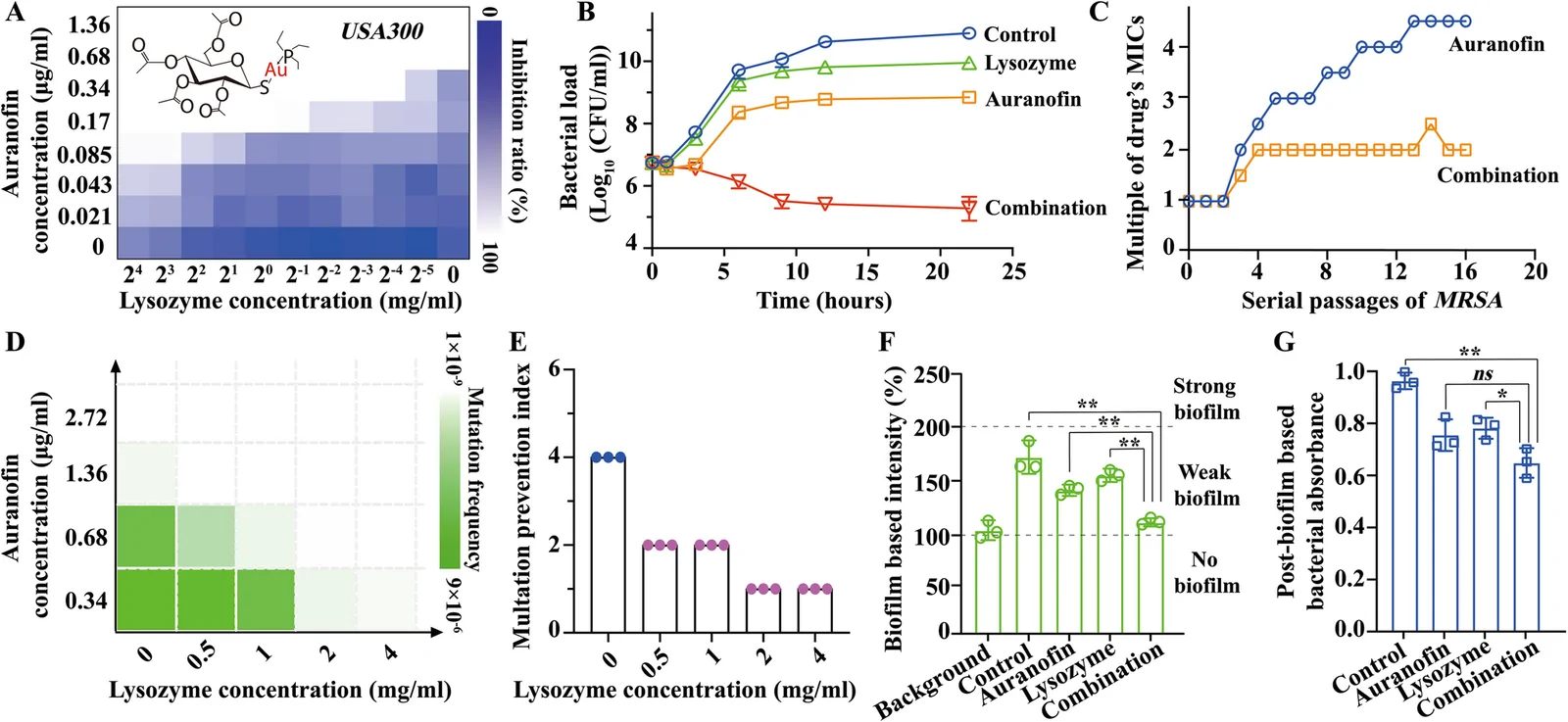
Combination therapy inhibited bacterial growth and resistance development. **(A)** Heat map illustrating USA300 response to combination therapy. The chemical structure of auranofin, a gold(I)-containing complex, is shown. **(B)** Time-kill curves depicting the growth of log-phase USA300 under different treatments. **(C)** Resistance acquisition curves over 16 passages in USA300 under different treatments. **(D)** Heat map displaying mutant frequencies in USA300 exposed to lysozyme, auranofin or their combination. (**E**) Mutation prevention index of auranofin when combined with lysozyme. **(F-G)** Crystal violet absorbance assays demonstrating the inhibition effects on USA300 under different treatments **(F)** before biofilm formation and **(G)** after biofilm formation. ^*^p < 0.05, ^**^p < 0.01 and ^***^p < 0.001

Combination therapy consisting of an antimicrobial and its adjuvant has been well documented as a safer and more economical strategy to re-sensitize ineffective antimicrobials [12–18]. This approach can also effectively reduce the clinical dosage of antimicrobials, thereby slowing the emergence of AMR-superbugs [1]. To find these adjuvants, the repurposing strategy of “new uses for old drugs with known safety profiles and pharmacokinetics” is gaining popularity because it facilitates rapid evaluation of newly identified combination therapies in phase II clinical trials [19]. A typical example is auranofin, which was repurposed as an adjuvant of colistin to restore its sensitivity in resistant bacteria [20]. It is a gold(I)-based complex where the gold ion is coordinated by sulfur and phosphorus ligands (Fig. 1A) [16]. Upon bacterial uptake, auranofin is metabolized to species such as triethylphosphine gold(I)^21^. Its primary antibacterial action arises by potently inhibiting thioredoxin reductase, thereby disrupting redox homeostasis and causing the accumulation of ROS [21]. This target is particularly crucial in Gram-positive bacteria, like MRSA.

In this report, we demonstrated that auranofin synergizes with lysozyme against diverse *Staphylococcus aureus* strains, including MRSA, based on compound screening results. By employing multiple technologies, we explored the mechanisms underlying this synergistic effect and highlighted the potential of auranofin as a novel adjuvant to potentiate the body’s own innate immune defenses to combat resistant bacteria.

## Materials and Methods

1. Bacterial strains, compounds, reagents and instruments. Methicillin-resistant *Staphylococcus aureus* USA300 (ATCC BAA-1556), methicillin-resistant *Staphylococcus aureus* USA100 (NRS382), methicillin-susceptible *Staphylococcus aureus* Newman strain (ATCC 25904) and ARPE-19 cell were from our own collection. Human lysozyme, ampicillin, 4-hydroxyethyl piperazine ethanesulfonic acid (HEPES), formaldehyde, crystal violet, sodium chloride (NaCl), Luria-Bertani (LB) broth powder and phosphate buffer saline (PBS) were supplied by Sigma-Aldrich. Auranofin was obtained as the ready-made solution (14.7 mg/ml in dimethyl sulfoxide (DMSO), MedChemExpress, China), consistent with its high solubility in DMSO (> 100 mg/ml)16. For all related assays, it was diluted to an intermediate concentration of 2 mg/ml in DMSO, then further diluted in sterile water to the final working concentrations, ensuring the final DMSO concentration was equal across groups and never exceeded 1% (v/v). Vehicle controls containing the corresponding DMSO concentration were included in all related assays. Waldiomycin was ordered from Biorbyt. Bacterial incubator, cell culture incubator and plate-reader were purchased from Thermal Fisher Scientific, USA. Deionized (DI) water (18.2 MΩ.cm) and filters (0.45 and 0.22 μm) were purchased from Millpore, USA. All other chemicals were purchased from MedChemExpress (China) unless otherwise stated.
2. Compound screening. 40 compounds (30 natural compounds and 10 metallodrugs from our drug library, fixed concentrations of 0.5 µM) were screened against USA300 in the absence and presence of 1 mg/ml human lysozyme. Specifically, these compounds were added into the 96-well microliter plate containing 5 × 106 colony-forming units per milliliter (CFUs/ml) bacterial suspensions. These wells were pre-contained with 50 µl of LB medium with or without 1 mg/ml lysozyme. LB medium without bacteria served as background group. Then, the real-time growth curves of USA300 were monitored per hour. Assays were performed in triplicate. By analyzing the optical density at 600 nm (OD600) of bacterial culture at 24-hour, the combination inhibition ratio (%) was calculated according to (ODcompound alone –ODcombination)/(ODcompound alone−ODbackground)×100%.
3. Checkerboard broth micro-dilution assays. According to two-fold dilution method []. Briefly, FICI= MICAB/MICA+MICBA/MICB =FICA+FICB. MICA is the MIC of compound A alone; MICAB was the MIC of compound A in combination with compound B; MICB was the MIC of compound B alone; MICBA was the MIC of compound B in combination with compound A; FICA was the FIC of compound A; and FICB was the FIC of compound B. Herein, synergy, part synergy and non-synergy were defined as FICI < 0.5, 0.5 ≤ FICI < 1 and FICI ≥ 1, respectively. Assays were performed in triplicate.
4. Time-killing curves. According to standard method [15], overnight-cultured USA300 was diluted and aliquoted into new sterile 50 ml tubes at 1:1000 ratios. Notably, compared with the checkerboard assay conducted under static incubation, the time-kill experiments involved shaking cultivation of bacterial suspensions, which provides favorable growth conditions such as enhanced aeration and homogeneous nutrient distribution, thereby promoting more vigorous bacterial growth, which can consequently increase microbial tolerance to antibiotics [22]. In this context, to ensure that the synergistic effects were clearly demonstrated under these more challenging dynamic conditions, the higher concentrations of compounds were employed in the time-kill studies. Specifically, the bacterial suspension was cultured in LB medium containing 0.34 µg/ml auranofin, 0.5 mg/ml lysozyme or their combination at 37 °C with shaking at 250 rpm. Bacterial suspension without any drugs served as control group. To measure their kinetic time-killing curves, 20 µl of each culture was extracted at time intervals of 0-, 2-, 4-, 6-, 9-, 12-, and 22- hour for measurement of the OD600 and CFUs. 10 µl of each dilution was spotted on the LB-agar plates and incubated at 37 °C for 24 h. All assays were performed in triplicate.
5. Resistance development study. USA300 at exponential phase was diluted (1:1000) into fresh LB medium containing different concentrations of auranofin in the presence or absence of 0.5 mg/ml lysozyme. After first passage (one passage per day) at 37 °C, the MIC was determined by standard two-fold serial dilutions and defined as initial MIC. In this study, these initial MICs were determined to be 0.68 µg/ml for auranofin alone and 0.17 µg/ml for auranofin in the presence of lysozyme. They were served as the baseline concentrations (initial concentrations). Next, bacteria from the well with the highest drug concentration permitting growth were passaged into fresh LB medium but with higher concentration of compound for next passage. The process was repeated until 17th day. By comparing auranofin’s MIC, the fold-change in resistance to auranofin was calculated by dividing the MIC of the passaged population by its respective initial MICs. Assays were performed in triplicate.
6. Mutation frequency and prevention concentration analysis. According to previously described method [16], USA300 culture in log phase were collected and concentrated into bacterial suspension of about 1.0 × 1010 CFU/ml in PBS buffer. Then, 100 µl of diluted USA300 suspension was evenly applied onto agar plates with gradient auranofin concentrations (including 0.34, 0.68, 1.36 and 2.72 µg/ml) in the presence of lysozyme (including 0.5, 1, 2 and 4 mg/ml). Diluted bacterial culture on agar plates with auranofin alone served as control group. Initial diluted USA300 suspension was also cultured on agar plate without any additions to figure out accurate bacterial concentrations. After 48 h incubation at 37 °C, colony counts on agar plates were figured out. Herein, the bacterial mutation frequency was calculated as colony counts treated group/colony counts initial supplied group. Besides, the minimal concentrations of auranofin combined with lysozyme in plates without any colony were recorded as mutation prevention concentrations (MPC) in combination therapy. Corresponding quotients between MPC and auranofin itself MIC in control group were figured as “mutation prevention index”. All assays were performed in triplicate.
7. Biofilm assays. According to the previous method [7], biofilm inhibition was assessed by using an adherence assay on 96-well plate. Briefly, bacterial suspensions (5 × 106 CFU/ml) were exposed to 0.34 µg/ml auranofin in LB medium in the absence and presence of 0.5 mg/ml lysozyme. LB medium with or without bacteria served as control or background group, respectively. After overnight incubation at 37 °C, bacterial suspensions were removed and these wells were gently washed twice with sterile PBS to remove exclusively non-adherent bacteria. The adherent biofilms were fixed by using 95% methanol at 60 °C for 15 min. Then, biofilms were stained with 1% crystal violet (100 µl per well) at 37 °C for 15 min, followed by gently washing with PBS to remove redundant crystal violet. Finally, the dye was solubilized with 150 µl of 95% ethanol per well at 37 °C for 30 min. The optical density of solution in each well was measured at 590 nm using a micro-plate reader. Strong biofilm, weak biofilm and no biofilm were defined as “ODsample≥2ODbackground”, “ODbackground< ODsample< 2ODbackground” and “ODsample≤ODbackground”, respectively [1]. To evaluate the combination effect on post-biofilm, adherent bacteria were pre-separated from 24-hour bacterial medium without any drugs. Then, sterile LB medium containing 0.34 µg/ml auranofin with or without 0.5 mg/ml lysozyme were added into these wells for future 24-hour culture. Next, the absorbance (OD600) was measured to analyze the growth of the bacterial culture in the post-biofilm. All assays were performed in triplicate.
8. Scanning Electron Microscope (SEM) analysis. According to previously method [1], USA300 single colony was picked up and cultured in LB medium. After 24 h incubation at 37 °C, bacterial suspension was inoculated into LB medium supplied with auranofin (0.17–0.34 µg/ml), lysozyme (0.5 mg/ml) or their combination. After 5 h incubation at 37 °C, these bacteria in log phase were collected by centrifugation at 3220 *g* for 10 min and fixed with 2.5% glutaraldehyde prior to being washed and re-suspended in PBS. After that, bacterial cultures were dehydrated by using increasing concentrations of ethanol in water (10%, 30%, 50%, 70%, 80%, 90% and 100%) for 10 min in each step. Then, 5 µl of bacterial suspension at 1.0 × 109 CFU/ml was incubated on poly-ethylenimine-coated coverslips (22 mm×22 mm) and the coverslips were dried in a critical point dryer (Balzers, Liechtenstein, Germany) prior to mounting on 25 mm aluminum stubs with double-sided carbon tabs. The edges of the coverslips were treated with silver liquid, dried, and then gold-coated in an Edwards S150B sputter coater (Edwards High Vacuum, Crawley, West Sussex, UK). The cells were imaged with a TESCAN VEGA3 scanning electron microscope (TESCAN, Brno, Czech Republic) at a voltage of 20 kV.
9. Proteomics analysis. According to the previously described method [23], USA300 in log phase was treated with lysozyme (0.5 mg/ml) in combination of auranofin (0.17–0.34 µg/ml) at 37 °C. Those exposed to lysozyme (0.5 mg/ml) alone served as control groups. After 5 h incubation, cells were harvested and the total proteins of samples were extracted using the multiple freezing-thawing at liquid nitrogen followed by sonication. Then, BCA kit was used to quantify the extracted proteins for next digestion by trypsin at the ratio of 5:1. These overnight digested peptides were further labeled by TMT [10]plex before loading onto Orbitrap Exploris™ 480 Mass Spectrometer coupled with an UltiMate 3000 UPLC System with C18 analytical column. Mobile phases A and B consisted of 0.1% FA in water and 0.1% FA in 100% ACN, respectively. Mobile phase B was increased to 6% at 12 min, 20% at 82 min, 30% at 92 min, 90% at 100 min and held for 5 min. Data was collected in data-dependent acquisition (DDA) mode with HCD fragmentation at TopN mode. The resolution was set at 60,000 for MS1 and 15,000 for MS2 with 30 ms maximum injection time. All resulted spectra were searched against UniProt *Staphylococcus aureus* (20,330 entries, accessed 09/2019) using MaxQuant 1.5.8.2. The parameters for searching: a mass tolerance of 10 ppm for precursor ions; ±0.1 Da for fragment ions, carbamidomethylation on cysteine was set as a fixed modification, oxidation on methionine and protein N-terminal acetylation was set as variable modifications. The enzyme was specified as trypsin with two missed cleavages allowed. False discovery rate for peptide spectral matches and proteins was set as 1%. The maximum number of modifications per peptide was three. Differentially expressed proteins at protein-levels were identified with *p*-values ≤ 0.05 and fold change (FC) values ≥ 1.2 (log2FC ≥ 0.26 or log2 FC≤ -0.26). The cuff-diff program was used to analyze differences between two treatments. Similar protocol was performed in parallel reaction monitoring (PRM) analysis with minor revision [24]. Overnight digested label-free peptides were loaded onto Orbitrap Exploris™ 480 Mass Spectrometer. PRM acquisition methods were directly developed according to our DDA data. The DDA data was imported into Skyline to select targeted peptides from previously selected targets. The relative information including precursor *m/z*, charge, and retention time window of the selected peptides was exported from skyline into xcalibur software to edit the PRM method. The targeted MS1 parameters were as follows: resolution, 60,000; AGC target, 2.0 × 105; and maximum injection time, 50 ms. PRM scanning was performed at 30, 000 resolution, 1 × 105 AGC target, 54 ms maximum injection time and 0.7 *m/z* isolation window with peptides in 5 minutes retention time windows. After PRM data acquisition, the data was imported into skyline for analysis to confirm the confidence of proteomics analysis results. To get reliable identification and quantification, idotp and dotp were > 0.60. The top three product ions were summed up to represent the peptide abundance.
10. Bacterial ROS quantification. According to the described method [1], overnight bacterial cultures were 10-fold diluted into 200 µl of sterile LB medium black-walled 96-well plates containing 50 µM dihydrorhodamine 123 with or without 2% dimethylsulfoxide, lysozyme (0.5 mg/ml), and/or auranofin (0.34 µg/ml). Bacteria without any drug treatment served as no drugs group. Plates were incubated with no shaking at 37 °C for 3 h. Next, the OD600 was measured. Meanwhile, dihydrorhodamine 123 fluorescence (excitation at 507 nm, emission at 529 nm) was also measured to determine intercellular ROS accumulation. The ROS level was further normalized to OD600 to match differences in bacterial growth. All assays were performed in triplicate and results were expressed as average ± SD.
11. ATP level. According to the described method [23], USA300 in log phase was treated with lysozyme (0.5 mg/ml), auranofin (0.34 µg/ml) or their combination at different concentrations. Those without any treatment served as control group. After 1 h incubation at 37 °C, all bacteria were collected and washed by cold PBS for 3 times. Then, these collected samples were further re-suspended in cold extracting buffer (Beyotime, China) to get bacterial suspension with an initial density of about 5.0 × 106 CFU/ml, followed by sonication lysis for 40 min at 4 °C and centrifugation at 18, 000 *g* at 4 °C for 20 min to collect these supernatants. Then, 20 µl of each supernatant were added white 96-well plate pre-reactivated by 100 µl of ATP test solution to perform luminometer measurement. All assays were performed in triplicate and results were expressed as average ± SD.
12. RNA isolation and RT-qPCR . According to the reported method [25, 26], the total RNA was extracted from USA300 under different treatments for 1 h by using TRIzol kit and RNeasy minelute kit. Reverse transcription was conducted with 1 µg of total RNA using the goldenstarRT cDNA synthesis mix kit. Then, the real-time quantitative polymerase chain reaction (RT-qPCR) was performed using the SYBR master mix. Relative levels of genes expression were quantified using the comparative CT method, where the CT values were normalized with the housekeeping gene *rpoB* for comparison [27].
13. Cell-based infection model and cytotoxicity analysis. According to the described method [15, 16], ARPE-19 cell was firstly cultured in Gibco Dulbecco’s modified eagle medium: nutrient mixture F-12 (DMEM/F12) supplemented with 10% fetal bovine serum (FBS) and grown at 37 °C in 5% CO2-humidified atmosphere for 7 days. Then, about 1.0 × 104 collected ARPE-19 cells were seeded into per well of 96-well plates and incubated overnight to ensure their confluences. Logarithmic cultures of USA300 were washed with PBS for three times and re-suspended in DMEM/F12-10% FBS to get bacteria suspensions with initial density of about 5.0 × 106 CFU/ml. Next, 10 µl of bacterial suspension were added to each well and incubated for another 3 h (the multiplicity of infection (MOI) of 5). Next, infected cells were washed vigorously with PBS for six times and replenished with culture medium to remove unbound bacteria. Herein, cell-associated bacteria were defined as bacteria that attach to, penetrate, or transcytose in ARPE-19 cells. Next, these ARPE-19 cells were exposed to either lysozyme, auranofin or their combination overnight under identical cell culture condition (100 µl of per well). Uninfected cells in the absence of drugs served as positive control group. Infected cells without any drug treatment served as negative control group. After 24 h incubation, CCK-8 assays were performed to analyze the survival ratio of cells under different treatments. Specifically, 10 µl of CCK-8 solution per well was added and these cells were incubated for another 2 h at 37 °C for chromogen development. The absorbance was measured at 450 nm, with 630 nm as reference wavelength. Herein, survival ratio= (Abssample -Absnegative control)/(Abspositive control -Absnegative control)×100%. The bacterial loads were examined by lysing ARPE-19 cells with 1% Triton X-100 in PBS and serially diluting the resulting lysates to enumerate bacterial colonies on agar plates. To analyze the cytotoxicity of auranofin, a similar protocol was followed, although ARPE-19 was not infected with any bacteria. These assays were performed in triplicate, and results were expressed as average ± SD.

## Results

### Compound Screening Revealed that Auranofin Enhanced Antibacterial Activity of Lysozyme Against *Staphylococcus Aureus*

Using the standard methicillin-resistant *Staphylococcus aureus* USA300 (a MRSA strain, ATCC BAA-1556) as a model strain, we initially assessed the combination effects between lysozyme (a natural antimicrobial protein within body’s innate immune system [11]) and 40 bioactive compounds (30 natural compounds and 10 metallodrugs from our drug library) at fixed concentrations by monitoring bacterial growth curves over 24 h and observed a synergistic effect between lysozyme and auranofin, as indicated by a high combination inhibition ratio (> 90%, Table 1) and low FICI (0.266, Fig. 1A, S1A). Notably, the strain exhibited the MICs of ≥ 150 mg/ml for lysozyme and 0.17–0.68 µg/ml for auranofin (due to the inoculum effect) (Fig. S2). In this context, our study demonstrated that USA300 could be re-sensitized to lysozyme in the presence of 0.17 µg/ml auranofin, leading to a reduction of the lysozyme’s MIC level from ≥ 150 to 0.5 mg/ml (Fig. 1A).

**Table 1:** The initial screening for synergy identified auranofin as a promising candidate. The combination of auranofin with lysozyme (1 mg/ml) exhibited a significantly higher inhibition ratio (%) against USA300 than either agent alone, demonstrating a clear synergistic effect. In this screen, all tested compounds (10 metallodrugs labeled with underlines and 30 natural products) were sourced from our in-house library and used at a fixed concentration of 0.5 µM^a^. A combination inhibition ratio of ≥ 90% is highlighted in blue. All assays were conducted in triplicate, and data are presented as the mean ± SD

Compounds	Combination inhibition ratio (%)	Compounds	Combination inhibition ratio (%)
Andrographolide	1.12 ± 1.19	Artemisinin	24.33 ± 2.89
Asiatic acid	14.44 ± 3.09	*Auranofin*	97.78 ± 2.12
Berberine	10.06 ± 1.32	Betulin	27.46 ± 0.54
Betulinic acid	20.49 ± 0.17	*Bismuth subnitrate*	22.26 ± 2.06
Boswellic acid	30.66 ± 4.16	Brucine	12.19 ± 5.99
*Calcium dobesilate*	37.74 ± 0.75	Celastrol	58.2 ± 2.77
*Chromium picolinate*	19.07 ± 4.54	Chrysin	10.25 ± 3.22
*Cisplatin*	12.17 ± 16.65	Curcumin	15.87 ± 0.58
Curcusone B	17.91 ± 6.88	Fenbendazole	-12.48 ± 5.46
*Ferric maltol*	19.58 ± 12.36	*Gadoxetate*	18.69 ± 11.53
Galanthamine	18.18 ± 0.49	Ginsenoside	-12.99 ± 3.02
Isorhamnetin	38.55 ± 4.33	*Lithium clavulanate*	20.73 ± 8.45
Matrine	17.88 ± 1.15	Oleanolic acid	18.78 ± 3.82
*Oxaliplatin*	50.44 ± 9.93	Pinicolic acid A	8.38 ± 4.73
Resveratrol	36.06 ± 7.06	Rotundic acid	15.69 ± 5.55
Rutin	-16.55 ± 0.09	*Silver diethyldithiocarbamate*	21.45 ± 17.44
Spironolactone	6.26 ± 5.27	Strychnine	2.92 ± 6.47
Taccalonolide C	26.68 ± 4.32	Topotecan	28.89 ± 8.04
Triptolide	51.55 ± 5.45	Usnic acid	42.98 ± 6.63
Vincamine	-13.22 ± 0.43	Yamogenin	-27.55 ± 10.42

^a^The MICs of all screened compounds were measured and found to be not less than 1.0 µM. In the initial screening at 0.5 µM, most compounds did not significantly inhibit USA300 growth (OD_600_≥0.6) compared to the untreated control (OD_600_=0.6504 ± 0.0224). The only exceptions were auranofin (OD_600_=0.4507 ± 0.0528) and asiatic acid (OD_600_=0.2811 ± 0.0144)

To further confirm the effect of auranofin in enhancing lysozyme sensitivity, time-killing curve was then drawn [15, 28]. The results showed that their combination significantly reduced the bacterial load from the level of 10^10^ to 10^5^ CFU/ml (Fig. 1B, S1B). In contrast, auranofin or lysozyme alone showed only modest growth inhibition compared to the robust effect of the combination. Next, to determine whether the observed combination effect was broadly applicable against *Staphylococcus aureus* with diverse genetic backgrounds, we further performed the checkerboard analyses on additional two representative strains, i.e., the methicillin-resistant USA100 (NRS382, a predominant hospital-associated MRSA clone) and the methicillin-susceptible Newman strain (ATCC 25904, a model strain for host-pathogen studies) [29–32]. Our results demonstrated that the combination of auranofin and lysozyme exhibited synergistic effects against all newly tested strains, although the degree of synergy varied among them (Fig. S3). Due to the predominant role in global community-onset infections [33] and great combination effect, the MRSA strain USA300 was selected as the representative strain for all subsequent mechanistic and infection model studies.

Given the persistent challenge of resistance development in bacterial infection treatment [34–37], the impact of combination therapy on resistance development was assessed. However, due to the intrinsic lysozyme resistance of USA300, we thus focused our serial passage assay specifically on evaluating the potential for resistance development to auranofin in the absence and presence of lysozyme. Our results demonstrated that combination therapy effectively suppressed auranofin resistance development in USA300 if compared to auranofin monotherapy (Fig. 1C). Further investigations revealed that the gene mutation frequency of USA300 treated with 0.34 µg/ml auranofin significantly decreased from ≥ 9×l0^− 6^ to ≤ 1×l0^− 9^ when combined with 2 mg/ml lysozyme (a physiological concentration in tears) (Fig. 1D-E). These findings indicate that combination therapy could effectively minimize the emergence of resistance development.

The presence of bacterial biofilm significantly challenges the clinical efficacy of antimicrobials [7]. To determine whether biofilm formation was inhibited, we subsequently performed crystal violet assays on bacteria in early and late exponential phases [1]. Interestingly, while treatment with auranofin or lysozyme alone only slightly reduced biofilm-based absorbance at 590 nm, the combination therapy significantly reduced biofilm biomass, bringing it to a level comparable to the background group (Fig. 1F). However, this reduction was not observed once the biofilm had already formed (Fig. 1G), likely due to difficulties in drug penetration into the EPSs [7]. Overall, these findings indicate that the synergistic effect between auranofin and lysozyme effectively combats USA300.

## Multi-Technical Studies Revealed the Synergistic Mechanism Centered on Autolysis and Cellular Metabolic Crisis

**Fig. 2:**
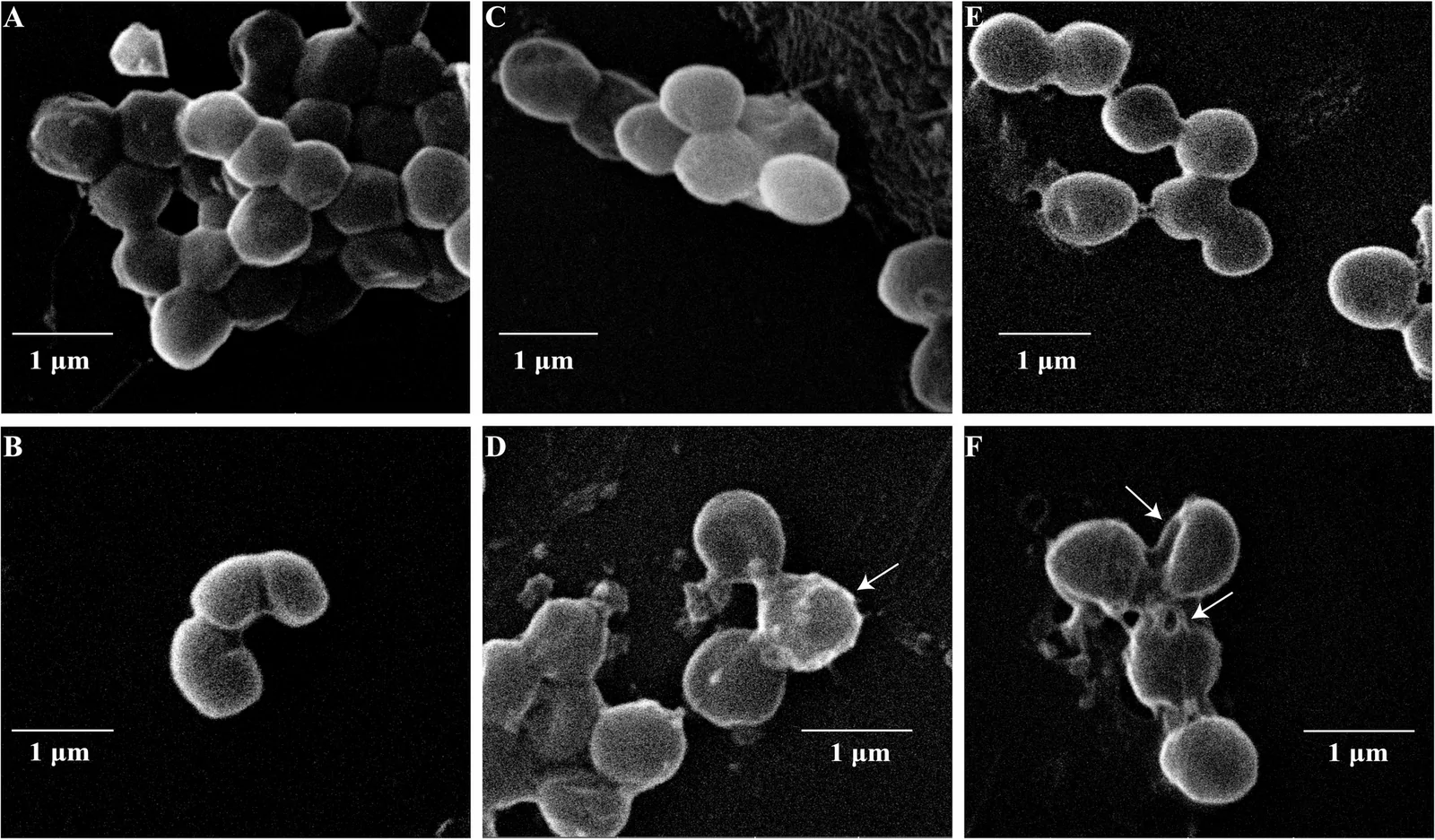
Morphological changes in USA300 with or without drug treatment. SEM images of **(A)** untreated USA300 and USA300 incubated with **(B)** 0.5 mg/ml lysozyme, **(C**,** E)** 0.17–0.34 µg/ml auranofin alone or **(D**,** F)** their combination for 5 h. White arrowheads indicated damaged cell walls and cell autolysis, including prominent membrane blebbing, pitting, rupture, and consequent cellular shrinkage and collapse. Representative images are presented

To explore how the combination therapy destroys USA300, we first conducted morphological analyses by comparing SEM images of USA300 treated with auranofin (0.17–0.34 µg/ml), lysozyme (0.5 mg/ml) alone, or their combination. As shown in Fig. 2 and S4, the cell surface of the untreated group appeared relatively smooth, a feature also observed in cells treated with auranofin or lysozyme alone. In contrast, USA300 treated with the combination therapy exhibited severe cell autolysis-like surface damages, including prominent membrane blebbing, pitting, rupture, and consequent cellular shrinkage and collapse. These morphological features suggested that bacteria might undergo severe cell autolysis under the combination treatment and these damages were so extensive that bacteria were unable to self-repair and ultimately died.

**Fig. 3:**
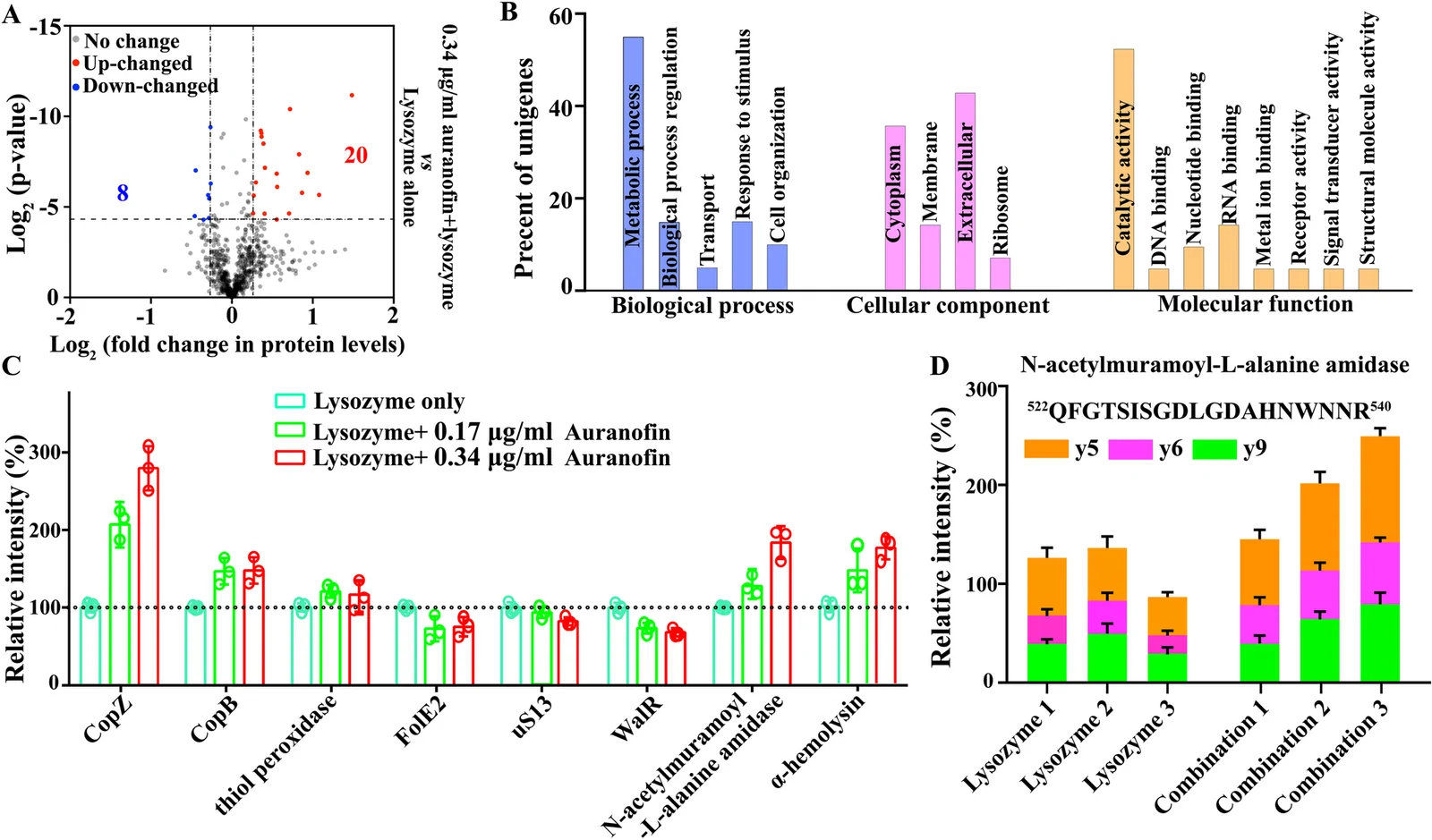
Proteomic analysis of USA300 treated with lysozyme in the absence or presence of auranofin. **(A)** Volcano plot and **(B)** Gene ontology annotation analysis of the differentially expressed proteins in USA300 exposed to lysozyme (0.5 mg/ml) with or without 0.34 µg/ml auranofin for 5 h. Dotted lines on the x- and y-axes represented an adjusted *p* value < 0.05 (Student’s t-test) and |Fold change| > 1.2, respectively. **(C)** Fold-changes of several notable proteins in USA300 under different treatments. (**D**) PRM analysis of N-acetylmuramoyl-L-alanine amidase (UniProt ID: Q2G222) in USA300 exposed to 0.5 mg/ml lysozyme with or without 0.34 µg/ml auranofin for 5 h. The inserted peptide represented the unique validated sequence in the PRM study

Subsequently, we performed a proteomic analysis on USA300 subjected to the different treatments to gain further mechanistic insights. Through the tandem mass tag (TMT) labeling for quantitative proteomics [24], we identified some up-regulated and down-regulated differentially expressed proteins (DEPs) in USA300 treated with lysozyme in the absence or presence of auranofin for 5 h (*p* < 0.05, Fig. 3A-B, S5). Gene ontology (GO) annotation analysis revealed that these DEPs were primarily associated with catalytic reactions, nucleotide binding, metabolic process, and regulation of biological process, and cytoplasmic localization (Fig. 3B, S5B).

**Fig. 4:**
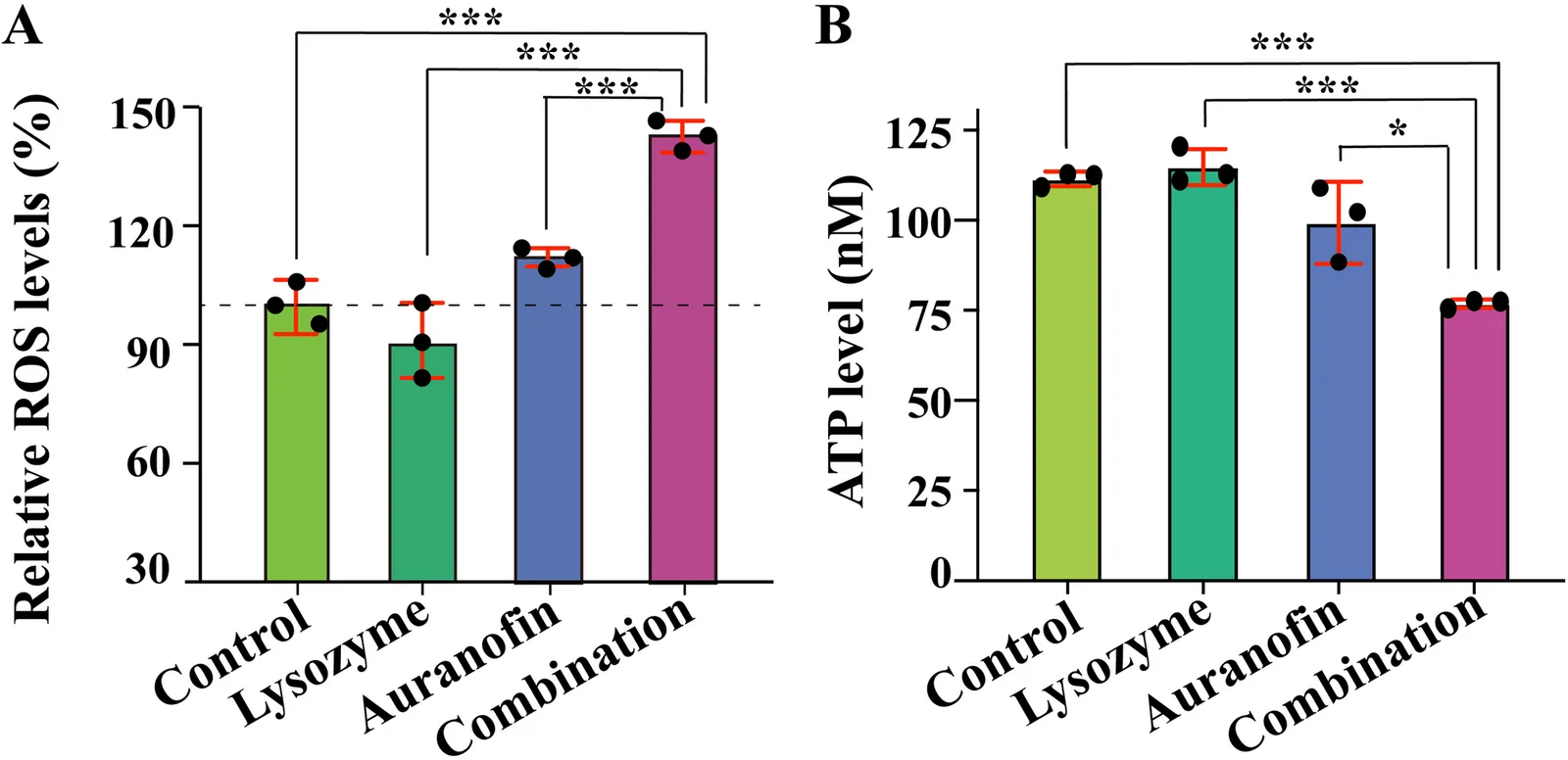
The combination therapy had significant effects on two critical cellular indices. The changes in (**A**) intercellular ROS accumulation and (**B**) ATP levels in USA300 with or without 0.5 mg/ml lysozyme treatments in the absence or presence of 0.34 µg/ml auranofin. All assays were performed in triplicate and results were expressed as average ± SD. ^*^p < 0.05, ^***^p < 0.001

Notably, several significant proteins exhibited remarkable changes when USA300 was treated with combination therapy (Fig. 3C, Table S1). A typical example was CopZ (UniProt ID: A6QK48, Fig. 3C), a chaperone involved in intracellular sequestration and transport of Cu^+^. Its over-expression (2.8-fold) was clearly observed in the combination therapy group. Besides, a similar overexpression was also found in the probable copper-transporting P-type ATPase B (CopB, UniProt ID: A8YZ02, Fig. 3C). Given the chemical similarity between Au^+^ and Cu^+^ ions^21^, these changes might be attributed to auranofin causing intracellular accumulation of metal (Au^+^) in USA300, leading to the overexpression of transporters for Cu^+ 38^. As a dominant enzyme against oxidative stress in Gram-positive pathogens, thiol peroxidase [16] (UniProt ID: Q5HF61, Fig. 3C) was overexpressed, likely in response to cellular stress induced by auranofin-mediated inhibition of thioredoxin reductase, which overwhelms cellular antioxidant defenses. However, this protective response was ultimately overwhelmed, which may have contributed to the catastrophic failure of cellular integrity. The intracellular level of ROS surged by 1.43-fold under combination treatment (Fig. 4A), which could potentially induce lipid peroxidation and thereby compromise membrane integrity. This oxidative assault may have been compounded by a concurrent energy crisis. Given the well-documented ability of auranofin to inactivate key enzymes in the glycolysis and tricarboxylic acid cycle (TCA) pathways [38], alongside the observed downregulation of GTP cyclohydrolase FolE2 (UniProt ID: Q2G0L1, an enzyme converting GTP to 7,8-dihydroneopterin triphosphate, Fig. 3C) and 30 S ribosomal protein uS13 (UniProt ID: Q6GEK7, Fig. 3C), these factors might collectively have contributed to a critical reduction in ATP levels (Fig. 4B).

Remarkably, our proteomic analysis revealed a critical disruption in the WalK/WalR two-component system, a master regulator of cell wall metabolism and autolysis in Gram-positive bacteria [39]. We observed a significant downregulation (nearby 0.68-fold) of the response regulator WalR (UniProt ID: Q7A8E1) in the combination therapy group (Fig. 3C). This dysregulation in WalR is of paramount functional importance, as the WalR protein acts as a transcriptional regulator of key autolysins [39]. Interestingly, our initial proteomic data also showed a pronounced increase (approximately 1.84-fold) in one of its potential direct targets, N-acetylmuramoyl-L-alanine amidase (UniProt ID: Q2G222, Fig. 3C). By analogy to well-characterized homologs (e.g.,* Escherichia coli* N-acetylmuramoyl-L-alanine amidase AmiA), this protein is inferred to cleave the bond between N-acetylmuramic acid and L-alanine in peptidoglycan, thereby acting as a potent autolysin from a bioinformatic perspective [40–42]. To robustly quantify these changes, we further performed parallel reaction monitoring (PRM) mass spectrometry (Fig. S6). PRM analysis confirmed the dysregulation of WalR and revealed a pronounced increase in this N-acetylmuramoyl-L-alanine amidase (Fig. 3D and S6F). Moreover, to directly assess WalRK regulon activation at the transcriptional level, we conducted RT-qPCR analysis. The results demonstrated a significant increase in the mRNA levels of two established WalRK target genes, *atlA* (encoding the major N-acetylmuramoyl-L-alanine amidase in MRSA) and *lytM* [43], upon combination treatment, thereby providing direct molecular evidence for the pathway’s activation (Fig. S7A-B).

Next, to functionally validate this role of WalRK two-component system in the synergy between lysozyme and auranofin, we used a pharmacological inhibitor waldiomycin [44], which could specifically block the activation of WalRK two-component system, to assess the impact on synergy. We found that the synergistic bactericidal effect of the combination was markedly attenuated (Fig. S7C). This result directly demonstrates that the dysregulation in the WalRK two-component system is essential for the full synergistic mechanism.

## Combination Therapy Effectively Reduced the Bacterial Load in a Cell-Based Infection Model

**Fig. 5:**
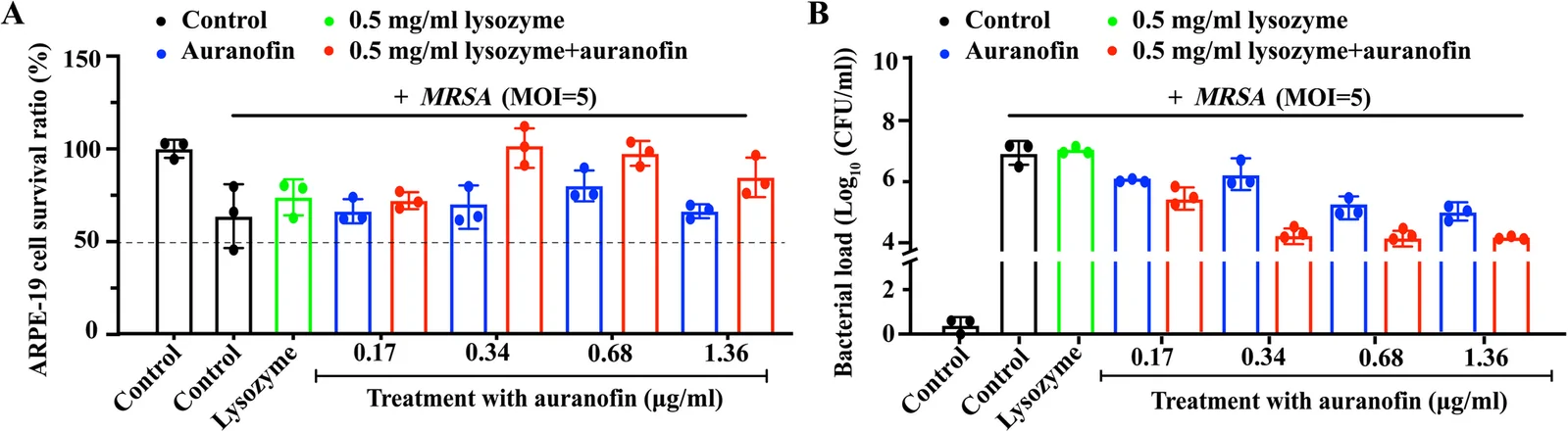
Auranofin and lysozyme exhibited a strong synergistic effect in the ARPE-19 cell-based infection model. **(A)** Survival ratio and **(B)** bacterial load of ARPE-19 cells were displayed. Overall, the combination significantly reduced the bacterial load and enhanced the survival ratio of ARPE-19 cells affected by USA300 infection

We next investigated the efficacy of combination therapy in eliminating USA300 in mammalian cells. Given the well-known abundance of lysozyme in healthy human tears [11, 45], we conducted a bacterial infection assay on ARPE-19 cells, a spontaneously derived retinal pigment epithelia cell line. Our results revealed that auranofin had minimal impact on ARPE-19 cells at the aforementioned concentrations (IC_50_~2.21 ± 0.34 µg/ml, Fig. S8). Remarkably, USA300 infection led to the death of about 35% ARPE-19 cells (Fig. 5A). Given that endogenous lysozyme production by ARPE-19 cells is primarily intracellular and its concentration (~ 25 µg/ml)^46^ falls below the threshold required for synergy with auranofin (≥ 62.5 µg/ml, Fig. 1A), we supplemented the culture medium with exogenous lysozyme (0.5 mg/ml), a concentration comparable to physiological levels in healthy human tears (~ 1 mg/ml)^45^, to effectively evaluate the combination’s efficacy in the infection model. Interestingly, with increasing concentrations of auranofin in the presence of 0.5 mg/ml lysozyme, we observed a higher survival ratio of infected cells (Fig. 5A) and a reduction in bacterial load (Fig. 5B) under the combination treatment. Specifically, the bacterial load significantly decreased to the level of 10^4^ CFU/ml (a 200-fold reduction) when ≥ 0.34 µg/ml auranofin was combined with 0.5 mg/ml lysozyme, and such reduction remained until the highest tested concentration of auranofin (1.36 µg/ml). However, increased death of infected ARPE-19 cells was also observed when higher concentrations of auranofin (≥ 0.68 µg/ml) were used in combination therapies, which might partly due to the fragile cellular state during bacterial infection and the elevated expression of α-hemolysin (a known pore-forming toxin that compromises eukaryotic cell membranes and can induce osmotic lysis [46], UniProt ID: Q2G1 × 0) as observed in our previous proteomics study (Fig. 3C, S6G).

## Discussion

In the post-antimicrobial era where the development of novel antimicrobials faces significant obstacles [16, 47], the rise of AMR pathogens is presenting a major challenge for clinical therapy [47, 48]. Interestingly, certain human tissues, e.g., eyes, are expected to possess robust defenses against bacterial infections through the secretion of natural antimicrobials, such as lacritin in tears [49], highlighting the critical importance of harnessing the body’s own immune defense system. Yet reality falls short of expectations. A stark example is the estimated 100 million patients worldwide who suffer annually from vision impairment caused by drug-resistant ocular infections [50].

Lysozyme, an essential component of the innate immune system, is gaining significant attention for its ability to kill Gram-positive bacteria by breaking down their cell walls [11]. This antimicrobial activity is known to be potentiated by various antibiotics, forming synergistic combinations. For instance, lysozyme has been reported to enhance the efficacy of metronidazole by increasing the permeability of the bacterial cell membrane in *Helicobacter pylori*, and to facilitate the entry of linezolid by disrupting the bacterial cell wall in MRSA [51]. This study demonstrates that a combination of auranofin and lysozyme can inhibit bacterial growth, offering a promising approach to combat drug-resistant bacterial infections by reinvigorating the host’s inherent defense mechanisms. In addition, the use of lysozyme, a natural component of innate immunity, offers practical advantages in resource-limited settings due to its accessibility and stability. While auranofin is an FDA-approved drug for rheumatoid arthritis [16], its antimicrobial efficacy in combination with lysozyme is constrained by a narrow therapeutic window against mammalian cells, as reflected in the low IC_50_/MIC ratio observed here. This limitation underscores the necessity to improve selectivity through strategies such as targeted delivery or optimized dosing regimens. Future studies could also investigate whether this strategy can be extended to potentiate other host defense molecules (e.g., defensins) against a broader spectrum of resistant pathogens.

Our proteomics-driven mechanistic investigation indicated that this combination dysregulates the WalR and its potential downstream autolysin, N-acetylmuramoyl-L-alanine amidase in USA300. This finding strongly implicates cell autolysis as a key pathway underlying the synergy with lysozyme. Meanwhile, the combination likely elevates WalR phosphorylation, which dictates its activity as transcriptional regulator even as its total abundance is reduced. Moreover, ATP reduction may further exacerbate bacterial death. Such energy deficit would cripple essential repair processes, thereby synergizing with increased ROS to exacerbate lethal damage. In this context, uncontrolled ROS accumulation might induce lipid peroxidation and membrane integrity, manifesting as surface blistering, pitting, and localized rupture. Subsequent leakage of cytoplasmic constituents (e.g., ions, metabolites, and ATP) through compromised membranes could disrupt osmotic homeostasis, potentially leading to the observed cellular shrinkage and structural collapse. In this context, the rationale for combining auranofin with lysozyme might stem from a strategy of attacking bacterial integrity both externally and internally. Lysozyme externally hydrolyzes the peptidoglycan layer, while auranofin internally facilitates autolysis, disrupts redox homeostasis and energy metabolism.

Beyond the aforementioned bacterial pathway, a potential direct physicochemical interaction could also be hypothesized. Based on a previously reported study [52], the interaction between the intact auranofin molecule and lysozyme involves only noncovalent interactions, forming a supramolecular adduct in which the drug occupies a hydrophobic pocket and does not involve direct gold-protein coordination, despite the high affinity of its gold metabolites for key amino acid residues in lysozyme, specifically cysteine (thiol groups), histidine (imidazole group), and methionine (thioether group) [16, 53]. Such binding may alter the spatial conformation and electrostatic environment of lysozyme, potentially enhancing its stability or lytic activity, thereby contributing to the observed antibacterial synergy. This hypothesis also offers a coherent explanation for the loss of efficacy against mature biofilms, as the resulting complex would be even more severely hindered by the EPS diffusion barrier than individual molecules despite bactericidal activity is also likely a major contributor to the reduced biofilm biomass. Nevertheless, the weak, noncovalent nature of the binding, which was notably absent for various bioactive gold metabolites of auranofin in the same study, coupled with the potential sequestration of the drug, may ultimately impede its cellular uptake and subsequent intracellular mechanisms of action. Consequently, the net contribution of this direct interaction to the overall antibacterial synergy is likely limited, although additional evidence is required. Despite this, it still indicates a potential to enhance combination effects through the development of an appropriate drug delivery system in the future.

Notably, the requirement for exogenous lysozyme supplementation in our cellular infection model merits explicit discussion, as ARPE-19 cells are recognized producers of this enzyme [54]. While endogenous lysozyme is present intracellularly at concentrations approximately reaching 25 µg/ml, its secreted level under standard culture conditions is negligible. Critically, this basal amount falls below the minimum threshold required to achieve synergistic bactericidal activity with auranofin, as shown in our checkerboard assays. Furthermore, the concentration used in our model aligns with physiological levels found in human ocular fluids, such as tears [45]. Therefore, the addition of exogenous lysozyme was necessary to attain a therapeutically relevant concentration capable of effective synergy with auranofin, ensuring a robust evaluation of the combination’s protective efficacy in the host cell context.

Collectively, we demonstrated that the in vitro antimicrobial efficacy of combination therapy could be well-translated into cellular efficacy. However, given the significance and complexity of in vivo studies, further study is needed to validate this approach before considering potential clinical applications.

## Supplementary Information

Below is the link to the electronic supplementary material.

Supplementary Material 1

Supplementary Material 2

Supplementary Material 3

Supplementary Material 4

Supplementary Material 5

Supplementary Material 6

Supplementary Material 7

Supplementary Material 8

Supplementary Material 9

Supplementary Material 10

## Data Availability

All assays were performed in triplicate and results were expressed as average ± SD unless otherwise stated. All tests of significance were based on ^*^*p* < 0.05, ^**^*p* < 0.01 and ^***^*p* < 0.001.All data related to this study are included in the paper or the supplementary material. Requests for material should be addressed to the corresponding author.
