# Supplementary material for: Synergistic Effects of Auranofin and Lysozyme Against Staphylococcus Aureus
Source: Probiotics Antimicrob Proteins. 2026 Mar 17;18(6):7668–80. doi: 10.1007/s12602-026-10983-2 (PMC13518490; doi:10.1007/s12602-026-10983-2)
Supplement: Supplementary file 1 [file 12602_2026_10983_MOESM10_ESM.docx]

***Supplementary Information For***

**Synergistic effects of auranofin and lysozyme against *Staphylococcus aureus***

Qi Zhang^1,2^, Yang Yang^1,2^, Shuqi Li^1^, Jiachi Chiou^3^, Qian Zhao^1,2*^

^1^Department of Applied Biology and Chemical Technology, The Hong Kong Polytechnic University, Hong Kong, China

^2^Centre for Eye and Vision Research (CEVR), 17W Hong Kong Science Park, Hong Kong, China

^3^Centre Department of Food Science and Nutrition, Research Institute for Future Food, The Hong Kong Polytechnic University, Hong Kong, China

^*^Corresponding author: Qian Zhao (email: [q.zhao@polyu.edu.hk](mailto:q.zhao@polyu.edu.hk))

**Fig. S1** Synergistic activity of auranofin and lysozyme against USA300. **(A)** Fractional inhibitory concentration (FIC) curves demonstrating the interaction between auranofin and lysozyme. **(B)** Representative images illustrating the inhibitory effect on USA300 growth when treated with 0.5 mg/ml lysozyme, 0.34 µg/ml auranofin individually, or in combination**.**

**Fig. S2** Inoculation density influenced the MIC_90_ of auranofin against USA300. Interestingly, USA300 showed resistance to lysozyme irrespective the inoculation density in this study. All assays were performed in triplicate.

**Fig. S3** Heatmaps showing synergistic activity of the auranofin-lysozyme combination against two *Staphylococcus aureus* strains, *i.e.,* **A**: USA100 (FICI=0.3125) and **B**: Newman strain (FICI=0.375).

**Fig. S4** Morphological changes in USA300 under different treatment. Lower-magnification SEM images of (A) untreated USA300 and USA300 incubated with (B) 0.5 mg/ml lysozyme, (C, E) 0.17 or 0.34 µg/ml auranofin alone or (D, F) their combination for 5 hours. Representative images are presented.

**Fig. S5** Proteomic analysis of USA300 exposed to lysozyme in the absence or presence of 0.17 µg/ml auranofin. **(A)** Volcano plot and **(B)** Gene ontology annotation analysis of the differentially expressed proteins in USA300 exposed to 0.5 mg/ml lysozyme in the absence or presence of 0.17 µg/ml auranofin for 5 hours. Dotted lines on the x- and y-axes represented an adjusted *p* value < 0.05 (Student’s t-test) and |Fold change| >1.2, respectively.

**Fig. S6** PRM analysis validated changes in (**A**) copper chaperone CopZ (UniProt ID: A6QK48), (**B**) probable copper-transporting P-type ATPase B CopB (UniProt ID: A8YZ02), (**C**) thiol peroxidase (UniProt ID:Q5HF61), (**D**) GTP cyclohydrolase FolE2 (UniProt ID:Q2G0L1), (**E**) 30S ribosomal protein uS13 (UniProt ID: Q6GEK7), (**F**) transcriptional regulatory protein WalR (UniProt ID: Q7A8E1), and (**G**) α-hemolysin (UniProt ID: Q2G1X0) in protein levels. The inserted peptide in each protein map was validated as a unique peptide in the PRM study.

**Fig. S7** Validation of WalRK pathway activation and its functional role in the synergy. (A) RT-qPCR analysis showing the transcriptional upregulation of two established WalRK target genes (*atlA* and *lytM*) upon combination treatment. (B) Sequences of the primer pairs used for RT-qPCR. (C) Waldiomycin, a pharmacological inhibitor of the WalRK two-component system, attenuated the synergistic bactericidal effect (FICI=0.75).

**Fig. S8** The toxicity of auranofin on ARPE-19 cells was analyzed using the CCK-8 kit. The calculated IC_50_ value for auranofin was determined to be 2.21±0.34 µg/ml.
